# Ground-Vegetation Clutter Affects Phyllostomid Bat Assemblage Structure in Lowland Amazonian Forest

**DOI:** 10.1371/journal.pone.0129560

**Published:** 2015-06-12

**Authors:** Rodrigo Marciente, Paulo Estefano D. Bobrowiec, William E. Magnusson

**Affiliations:** Instituto Nacional de Pesquisas da Amazônia, Coordenação de Biodiversidade, Manaus, Amazonas, Brazil; Università degli Studi di Napoli Federico II, ITALY

## Abstract

Vegetation clutter is a limiting factor for bats that forage near ground level, and may determine the distribution of species and guilds. However, many studies that evaluated the effects of vegetation clutter on bats have used qualitative descriptions rather than direct measurements of vegetation density. Moreover, few studies have evaluated the effect of vegetation clutter on a regional scale. Here, we evaluate the influence of the physical obstruction of vegetation on phyllostomid-bat assemblages along a 520 km transect in continuous Amazonian forest. We sampled bats using mist nets in eight localities during 80 nights (3840 net-hours) and estimated the ground-vegetation density with digital photographs. The total number of species, number of animalivorous species, total number of frugivorous species, number of understory frugivorous species, and abundance of canopy frugivorous bats were negatively associated with vegetation clutter. The bat assemblages showed a nested structure in relation to degree of clutter, with animalivorous and understory frugivorous bats distributed throughout the vegetation-clutter gradient, while canopy frugivores were restricted to sites with more open vegetation. The species distribution along the gradient of vegetation clutter was not closely associated with wing morphology, but aspect ratio and wing load differed between frugivores and animalivores. Vegetation structure plays an important role in structuring assemblages of the bats at the regional scale by increasing beta diversity between sites. Differences in foraging strategy and diet of the guilds seem to have contributed more to the spatial distribution of bats than the wing characteristics of the species alone.

## Introduction

The structure and composition of bat assemblages is mostly determined by vegetation features [1,2]. In particular, physical obstruction of the forest created by trunks, branches and leaves strongly effects habitat use by foraging bats directly because locomotion in cluttered spaces requires greater flight maneuverability [3,4]. Sites with very dense vegetation reduce foraging efficiency by limiting the movement of species and use of echolocation to detect obstacles and potential prey [5–9].

Phyllostomid bats are commonly captured in the understory of Neotropical forests and most species are frugivores, nectarivores, or gleaning animalivores [10]. These guilds forage in highly cluttered sites and collect food (fruits, nectar, insects, and small vertebrates) very close to vegetation [11]. In this situation, bats need specific sensory and morphological adaptations to fly in restricted space where food echoes may be hidden by clutter echoes [12].

For bats of the family Phyllostomidae, foraging strategies and wing morphology have been suggested to be the main factors associated with the ability to use cluttered environments [8, 12]. The different foraging strategies should reflect different use of space by bat species and guilds, and thus may determine the organization of bat assemblages. Gleaning animalivores capture their prey from ambush (sit-and-wait behavior) and depend on the sound generated by prey for localize the site with prey [12–14]. Because their wings are short, with a large surface area and rounded tips, gleaning animalivores have very maneuverable flight and can occupy sites with dense vegetation [8]. However, sites with highly cluttered vegetation hinder obstacle avoidance and may limit prey perception by echolocation [13–15]. Frugivorous and nectarivorous bats tend to be less agile in narrow spaces, but their long narrow wings allow a slower and efficient flight.

Foraging strategies vary between canopy and understory bats [16–18]. Canopy frugivores forage on trees that are usually patchily distributed and produce a great quantity of fruits for a relatively short period. These bats spend most of the night traveling long distances between areas in search of trees with ripe fruits. More open areas facilitate flight into the forest [19–21]. Understory frugivores consume fruits on shrubs and small trees with more localized distribution, which produce fewer ripe fruits per night, but with production extending over weeks or months. These species have shorter flights within a small area.

Processes that occur on a small spatial scale may play an important role in structuring assemblages at wider scales [22]. Microhabitat characteristics (e.g., canopy height, foliage structure) are known to influence tropical and temperate bat ensembles at intermediate to local scales [23–28]. The physical obstruction of vegetation imposes limitations on mobility and food detection by bats, changing the number and type of bat species that can coexist on a local scale. Therefore, it seems reasonable that vegetation clutter may also increase beta diversity between sites at regional scales.

Although vegetation clutter is known to exert a strong influence on the abundance and species composition of bats [1,2,4,29–31], many studies used qualitative descriptions of vegetation clutter (e.g. edge, open, structurally complex vs. simple), rather than direct measurements of vegetation density, and few studies have evaluated the effect of vegetation clutter at a regional scale [28]. In this study, we evaluate the influence of the physical obstruction of vegetation on Phyllostomid-bat assemblages at a regional scale, covering a 520 km transect in Central Amazonia. We hypothesized that vegetation structure, considered a useful predictor of the distribution of Phyllostomid bats at the local scale, also influences bat assemblages at a regional scale. We also assessed wing morphology of the species to test the relationship between species distribution along the gradient of vegetation clutter and wing morphology (aspect ratio and wing load) of the species. We expected that changes in assemblage composition would be mediated mainly by decrease in abundance and number of species in cluttered vegetation. Because of the different foraging strategy of the guilds, we also predicted that species would be distributed in a nested pattern along the gradient of vegetation clutter at a regional scale, with animalivorous and understory frugivorous bats present in both open and dense vegetation and canopy frugivores restricted to more open vegetation.

## Methods

### Ethics Statement

We followed the guidelines approved by the American Society of Mammalogists in our procedures [32]. All captured bats were handled by experienced an investigator. Captured bats were kept in cloth bags individually for the minimum possible time and were released at the place where they were captured. Voucher specimens were collected and deposited in the Mammal Collection of the Instituto Nacional de Pesquisas da Amazônia (INPA6418-INPA6726). Bat captures and handling were in accordance with Brazilian conservation and animal welfare laws, and was undertaken under scientific licenses from the Instituto Chico Mendes de Conservação da Biodiversidade—ICMBio (Permit numbers 25781–1 and 25799–2).

### Study area and Sampling Design

**Fig 1:**
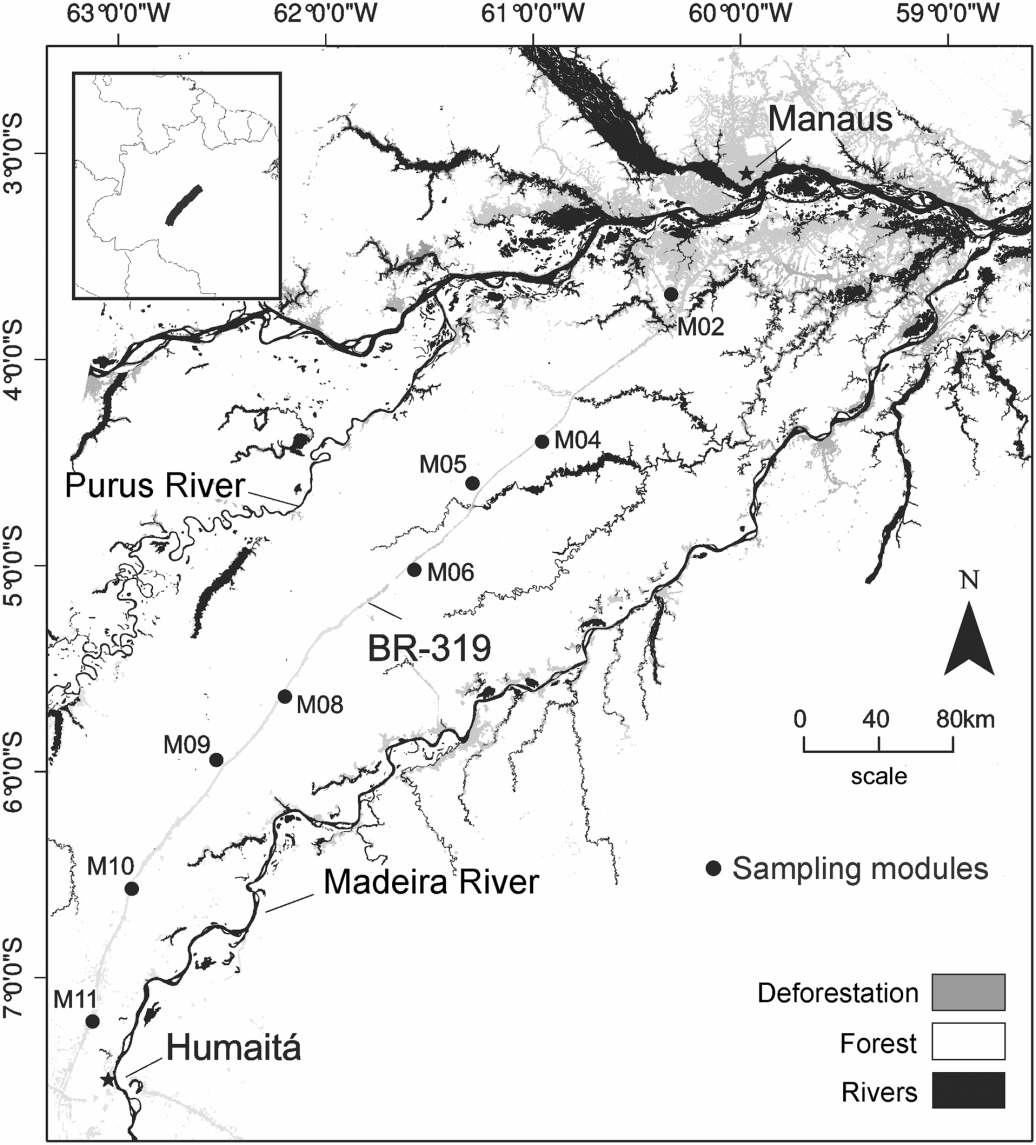
Map of the study area showing the eight sampling modules along the 520-km section of the BR-319 highway, Central Amazonia.

Bat assemblages were sampled along a 520-km section of the BR-319 highway that connects the municipalities of Manaus and Humaitá, between the Purus and Madeira Rivers in Central Amazonia (Fig 1). The vegetation is classified as dense tropical lowland forest in the northern part of the BR-319 highway and open tropical lowland forests in the southern part, near Humaitá [33]. In the central part of the interfluve, the maximum annual rainfall is around 2800 mm, while in the northern and southern parts, near Manaus and Humaitá, the maximum annual rainfall is around 2400 mm. The number of dry months (precipitation below 100 mm) per year varies from two to four months [34].

Bats were captured in 80 permanent plots distributed in eight sampling modules that are part of the Brazilian Biodiversity Research Program (PPBio; http://ppbio.inpa.gov.br). The modules were installed at intervals ranging from 40 to 60 km along the BR-319 highway (Fig 1). Each module consists of two 5 km parallel trails, with ten plots distributed at 1 km intervals, following the RAPELD sampling design [35,36]. Each plot was 250 m long and followed the altitudinal terrain contour in order to minimize variation in topography and soil properties, and consequently, variation in the vegetation type within each plot [35].

### Bat Captures

Bats were captured between October and November 2010 and between June and November 2011, during the dry season. We used eight mist nets (12 × 3 m, 19 mm mesh, Ecotone, Poland) installed at ground level along the center line of each plot. Nets remained open from 18:00 to 00:00 h and were checked at intervals of 30–45 minutes. Each plot was sampled for one night, totaling 10 capture nights and 480 mist-net hours (mnh) effort per module (one mnh denotes one 12-m net open for 1 h). Nights with full moon or strong rains were not sampled in order to minimize potential bias in capture success.

Bats were identified, measured and weighed before release. We identified bats using keys of Lim and Engstrom [37] and Gardner [38], supported by bat descriptions of Charles-Dominique et al. [39] and Simmons et al. [40]. Individuals of the genus *Carollia* were grouped as *Carollia* spp. because it was not possible to distinguish *C*. *perspicillata* and *C*. *brevicauda* based on external characters measured in the field. These species have the same foraging strategy and are frugivores [41–43]. Based on their habitat type, foraging mode, and echolocation behavior reported in the literature [12,16,30,43,44], species were categorized into six feeding guilds: cluttered-space passive-gleaning foragers (including gleaning insectivores and carnivores; hereafter denoted gleaning animalivores), cluttered-space passive/active-gleaning foragers (canopy frugivores, understory frugivores, and nectarivores), edge-space aerial foragers, and edge-space trawling foragers.

### Vegetation Clutter

We estimated the density of understory vegetation using digital photographs, adapted from Marsden et al. [45]. A white cloth mounted in a 3 × 3 m aluminum frame was placed 8 m from the central line of the plot and positioned perpendicular to a digital camera (S1 Fig). We recorded one photograph for each mist net, totaling eight photographs per plot and 80 photographs per module. We transformed the photos into black-and-white images using Sidelook 1.1.01 program [46], so that black areas represented vegetation. The vegetation clutter of each module was estimated as the average percentage of area covered by vegetation (trunks, branches, twigs, and leaves) in the 80 photographs.

### Data Analysis

We only analyzed data on bats of the family Phyllostomidae since species from other families are not adequately sampled with mist nets [11]. Only frugivores, gleaning animalivores, and nectarivores occur in the family. We evaluated inventory completeness with sample-based rarefaction curves based on species abundance randomized 1000 times [47]. In addition, we estimate the species richness using the Jackknife 1 estimator and compared it to the observed number of species to estimate the percentage of inventory completeness. Rarefaction curves and Jackknife 1 estimator were calculated with EstimateS software 9.1.0 [48].

An ordination by Non-metric Multidimensional Scaling (NMDS) was used to reduce the dimensionality of the species-occurrence data (presence-absence) in the modules to one ordination axis [49]. The dissimilarity between pairs of modules was calculated using the Sørensen index. We calculated the NMDS stress as a measure of goodness of fit to indicate how well the ordination preserves the original distance relationships among the samples [49]. Stress values < 0.2 are recommended [49]. We used generalized linear models (GLM) to evaluate the effect of vegetation clutter on the total number of species and abundance, number of species and abundance of the all frugivorous, canopy frugivorous, understory frugivorous, and animalivorous bats, and bat species composition (summarized by the single NMDS axis). The over dispersion of the residuals in the GLM models was controlled using a quasi-GLM regression model for richness and abundance of species and guilds [50].

**Fig 2:**
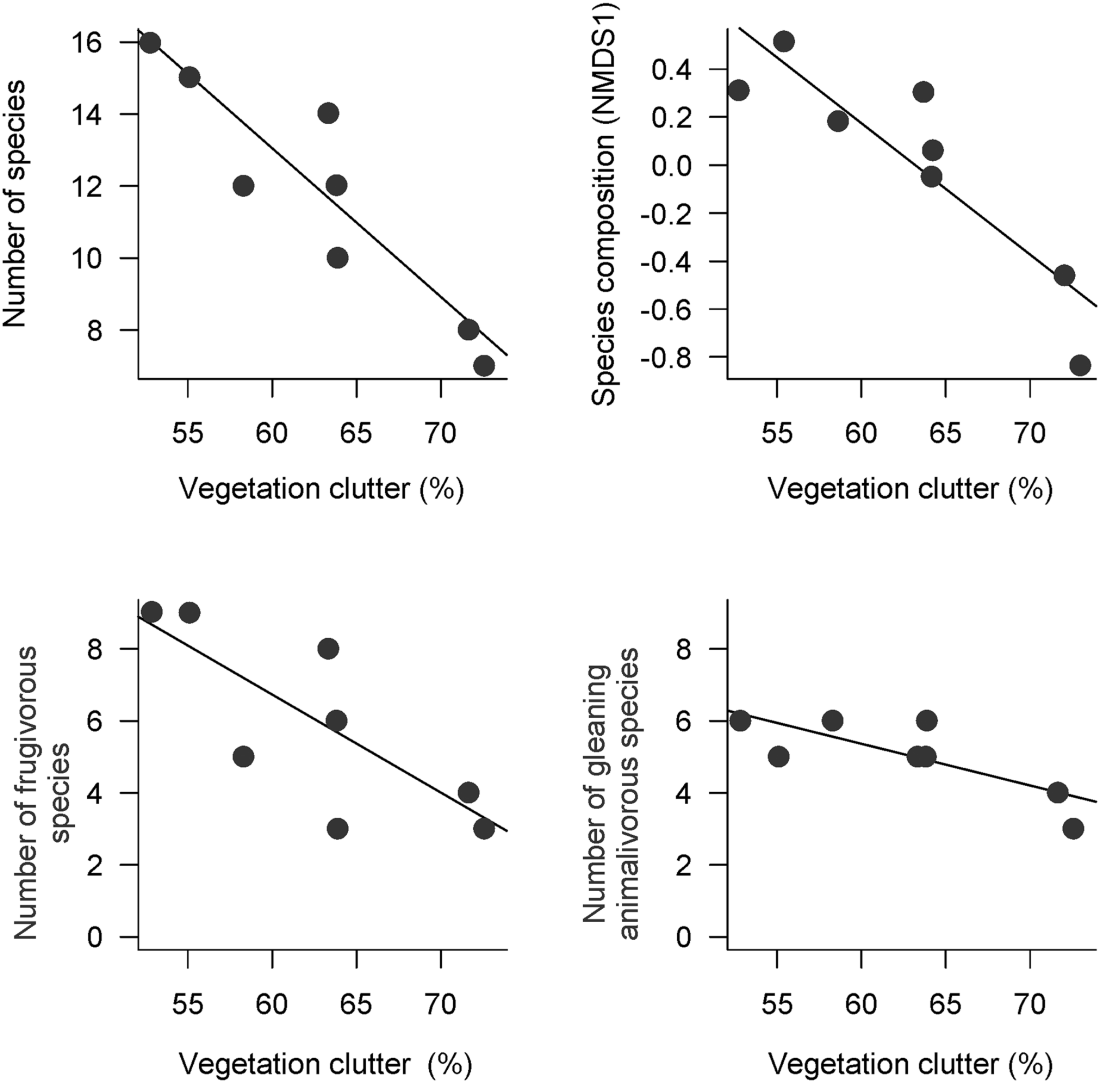
Relationship between bat abundance and the gradient of vegetation clutter. The horizontal order of the sampling modules was based on the gradient in vegetation clutter. The vertical order of species was based on the average number of captures weighted by vegetation clutter of each module, as indicated by rank values. Species with higher rank values are placed near the top of the graph. Black squares represent gleaning animalivorous bats, white squares canopy frugivores, grey squares understory frugivores, and hatched squares the nectarivore.

The species distribution in relation to differences in vegetation clutter between modules was evaluated by direct-gradient analysis (Fig 2). In this analysis, the modules were ordered in relation to the gradient in vegetation clutter. The species were ordered by the average number of captures weighted by vegetation clutter of each module, according to the formula:$Rank=\left(\sum [n_{ij}\times clutter_{j}]\right)/N_{i}$, where *nij* represent the number of captures of the species *i* in the module *j*, *clutter*_*j*_ is the average vegetation clutter value in module *j*, and *N*_*i*_ is the total number of captures of the species *i*. Higher values represent species captured in modules with more cluttered vegetation. The rank values of the species abundance weighted by the vegetation clutter of the animalivorous and all frugivorous species were compared with a *t* test, and differences among animalivorous, canopy-frugivorous, and understory-frugivorous species were compared using an Analysis of Variance (ANOVA) followed by a *post-hoc* Tukey test.

We evaluated whether the structure of the bat assemblages generated by direct-gradient analysis showed a nested pattern of species distribution among modules. Nestedness occurs when sites with lower species richness tend to harbor subsets of species present in richer sites. We applied the metric NODF (Nestedness Metric Based on Overlap and Decreasing Fill; [51]) with algorithm SIM2 [52] that is less prone to type I error in analysis of standardized "sample lists" of species collected in rapid ecological sampling with minor contribution of rare species. NODF is considered a robust metric for studies with low numbers of species, and to insensitive to shape and size of the data matrix [51]. We used a null model to evaluate whether the structure of the bat assemblages generated by direct-gradient analysis differs from a random distribution of species [53]. The probability was based on 10000 randomizations. As the NODF metric is dependent on the arrangement of columns and rows to allow testing hypotheses on the causes of nestedness, positions of species and modules in the null model were restricted to those of the direct gradient analysis (Fig 2) in which bat species were columns and modules were rows. The NODFc value quantifies the nested structure of the modules in relation to vegetation clutter.

To evaluate whether the species distribution along the gradient of vegetation clutter was associated with wing morphology, we related the rank values of the species abundance weighted by the vegetation clutter with wing characteristics (aspect ratio and wing load) using a quasi-GLM model. High values of aspect ratio and wing load indicate species with faster flight and lower maneuverability [8]. Aspect ratio and relative wing load were obtained from Marinello and Bernard [54] for bats of Central Amazonia. Aspect ratio and wing load of *Vampyressa brocki* were not found in the literature and the species was excluded from the analysis. The nectarivores were not included in the analysis because of the low number of captures and species. We compared aspect ratio and wing load between animalivorous and all frugivorous species with a *t* test, and between animalivorous, canopy frugivorous, and understory frugivorous species using an Analysis of Variance (ANOVA) followed by a *post-hoc* Tukey test.

Geographical autocorrelation of species abundance between pairs of modules may violate statistical assumptions of analyses and lead to inappropriate conclusions in studies that correlate species distribution with environmental factors [55]. We tested the spatial autocorrelation in the residuals of the GLM tests using Moran's I statistics. The distance classes were adjusted to intervals of 40 km (minimum distance between modules) and used an equal number of modules for each distance class. Moran's I statistic was tested for significance using 1000 permutations. We did not detect autocorrelation in the residuals of the GLM tests, indicating that the response variables of the modules were spatially independent.

Analyses were carried out with the R software version 3.1.1 [56]. The NMDS ordination and null model to test the significance of the nestedness pattern were undertaken in the R package ‘vegan’ [57]. The null model was generated with the oecosimu function and NMDS with the metaMDS function. Spatial autocorrelation analyses were performed in SAM software v. 4.0 [58]. The data and metadata are deposited in the public repository of the PPBio (http://ppbio.inpa.gov.br/repositorio/dados). To access the data use the key words "morcegos", "BR-319", and "clutter".

## Results

After 3840 mnh (80 nights of sampling), we captured 512 bats of 27 species from four families (Emballonuridae, Phyllostomidae, Vespertilionidae, and Thyropteridae) (Table 1). Phyllostomid bats accounted for most (98.2%) captures (Table 1). Most species (12) were frugivores and they accounted for most captures (n = 413), followed by gleaning animalivores (10 species and 70 captures) and nectarivores (1 species and 19 captures). Among the frugivorous bats, the three understory species accounted for 328 of the captures, while the canopy frugivores accounted for 86 captures distributed in nine species (Table 1). The number of phyllostomid species recorded in each module ranged from 8 to 16 (11.8 ± 3.2, mean ± SD) and the number of captures per module ranged from 13 to 137 bats (62.9 ± 46.3). The nine species with more than 10 individuals captured accounted for 89% of all captures. *Lophostoma silvicolum* was the only species captured in all modules, and *Artibeus concolor*, *Chrotopterus auritus*, *Rhinophylla fischerae*, and *Vampyriscus brocki* were each captured in only one module. *Carollia* spp. and *R*. *pumilio* were the taxa most captured and represented 34% and 33% of all captured individuals, respectively (Table 1). Species-accumulation curves and species-richness estimator (S2 Fig) indicated a high level (80%) of inventory completeness.

**Table 1:** Bats captured in eight modules along the BR-319 highway, Central Amazonia, Brazil.

Taxon	Captures	Range	Modules	Guilds
Emballonuridae				
*Saccopteryx bilineata*	1	0–1	1	Edge space aerial forager
Phyllostomidae				
Carollinae				
*Carollia* spp.	167	0–86	6	Cluttered space passive/active gleaning forager—understory frugivore
*Rhinophylla fischerae*	1	0–1	1	Cluttered space passive/active gleaning forager—understory frugivore
*Rhinophylla pumilio*	160	3–33	8	Cluttered space passive/active gleaning forager—understory frugivore
Lonchophyllinae				
*Lonchophylla thomasi*	19	0–7	7	Cluttered space passive/active gleaning forager—nectarivore
Phyllostominae				
*Chrotopterus auritus*	1	0–1	1	Cluttered space passive gleaning forager
*Lophostoma brasiliense*	2	0–1	1	Cluttered space passive gleaning forager
*Lophostoma silvicolum*	13	1–3	8	Cluttered space passive gleaning forager
*Micronycteris megalotis*	6	0–2	5	Cluttered space passive gleaning forager
*Mimon crenulatum*	6	0–2	4	Cluttered space passive gleaning forager
*Phylloderma stenops*	4	0–1	4	Cluttered space passive gleaning forager
*Phyllostomus elongatus*	13	0–4	5	Cluttered space passive gleaning forager
*Tonatia saurophilla*	8	0–2	5	Cluttered space passive gleaning forager
*Trachops cirrhosus*	15	0–5	5	Cluttered space passive gleaning forager
*Trinycteris nicefori*	2	0–2	1	Cluttered space passive gleaning forager
Stenodermatinae				
*Artibeus concolor*	1	0–1	1	Cluttered space passive/active gleaning forager—canopy frugivore
*Artibeus gnomus*	18	0–7	5	Cluttered space passive/active gleaning forager—canopy frugivore
*Artibeus lituratus*	4	0–2	3	Cluttered space passive/active gleaning forager—canopy frugivore
*Artibeus obscurus*	31	0–18	7	Cluttered space passive/active gleaning forager—canopy frugivore
*Artibeus planirostris*	12	0–6	3	Cluttered space passive/active gleaning forager—canopy frugivore
*Mesophylla macconnelli*	6	0–3	2	Cluttered space passive/active gleaning forager—canopy frugivore
*Uroderma bilobatum*	4	0–2	3	Cluttered space passive/active gleaning forager—canopy frugivore
*Vampyriscus bidens*	9	0–3	6	Cluttered space passive/active gleaning forager—canopy frugivore
*Vampyriscus brocki*	1	0–1	1	Cluttered space passive/active gleaning forager—canopy frugivore
Vespertilionidae				
*Myotis* sp.	3	0–2	2	Edge space trawling forager
Thyropteridae				
*Thyroptera discifera*	1	0–1	1	Edge space aerial forager
*Thyroptera tricolor*	4	0–2	3	Edge space aerial forager
Total	512	13–137		

**Fig 3:**
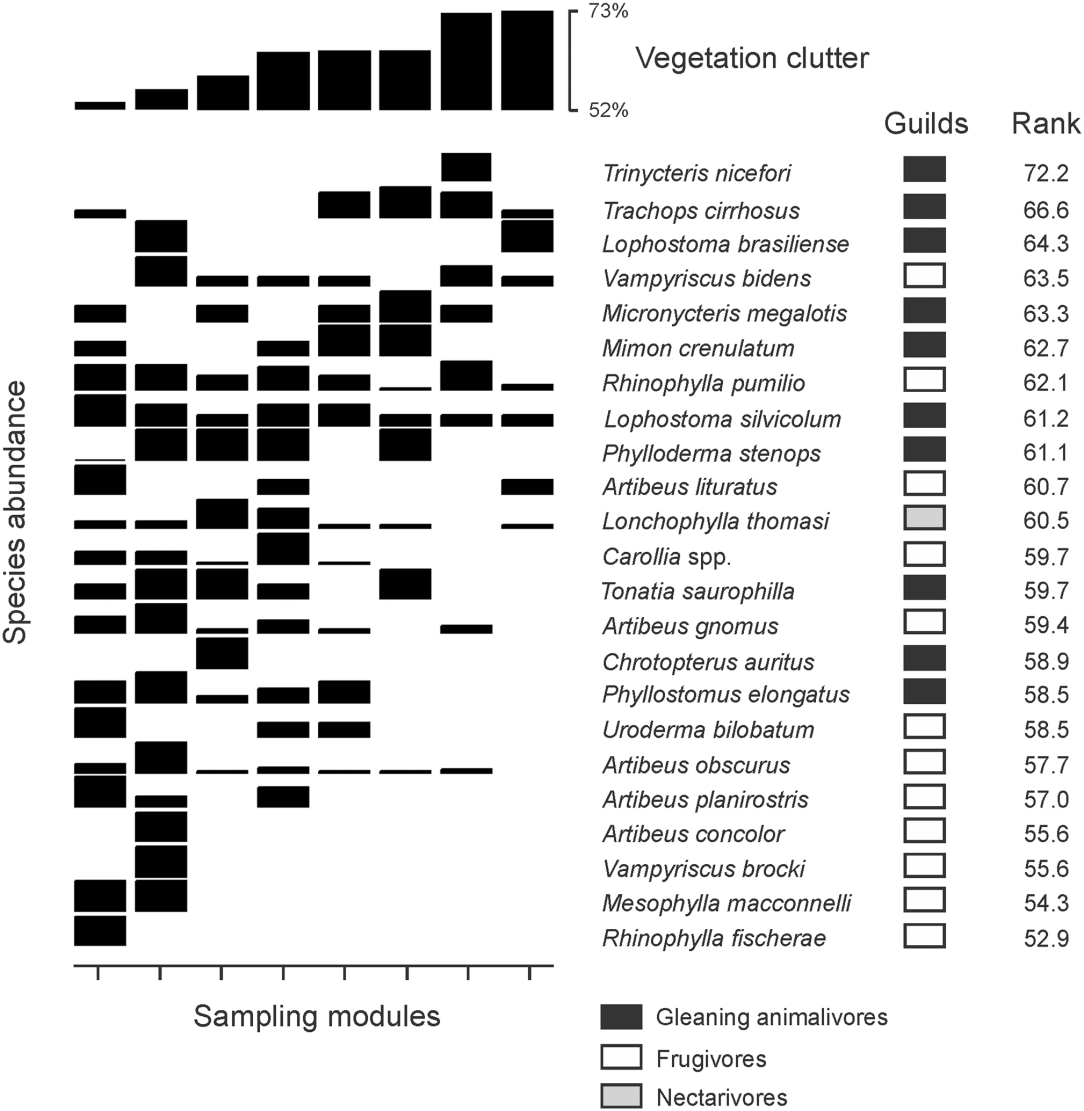
Relationships between vegetation clutter and (A) number of bat species, (B) bat-species composition summarized by the first axis of a NMDS analysis, (C) number of gleaning animalivorous species, (D) number of frugivorous species, (E) number of understory frugivorous species, and (F) abundance of canopy frugivores.

Ground vegetation clutter of the modules ranged from 52.9 ± 14.4% to 73 ± 12.8% (Fig 2). Vegetation clutter (Fig 3) was negatively associated with the total number of bat species (GLM, *r* ^*2*^ = 0.82, *t* = -5.12, *P* = 0.002), number of animalivorous species (GLM, *r* ^*2*^ = 0.57, *t* = -2.84, *P* = 0.030), number of frugivorous species (GLM, *r* ^*2*^ = 0.60, *t* = -2.98, *P* = 0.025), number of understory frugivorous species (GLM, *r* ^*2*^ = 0.79, *t* = -4.76, *P* = 0.003), and abundance of canopy frugivorous bats (GLM, *r* ^*2*^ = 0.55, *t* = -2.55, *P* = 0.044). Abundance of all species, all frugivores, and understory frugivores and number of canopy frugivorous species were not significantly influenced by vegetation clutter (*P* > 0.084, *r* ^*2*^ < 0.40). The NMDS ordination axis (Fig 3) explained 76% of the total variation (NMDS stress = 0.17) and was associated with vegetation clutter (GLM, *r* ^*2*^ = 0.79, *t* = -4.72, *P* = 0.003).

**Fig 4:**
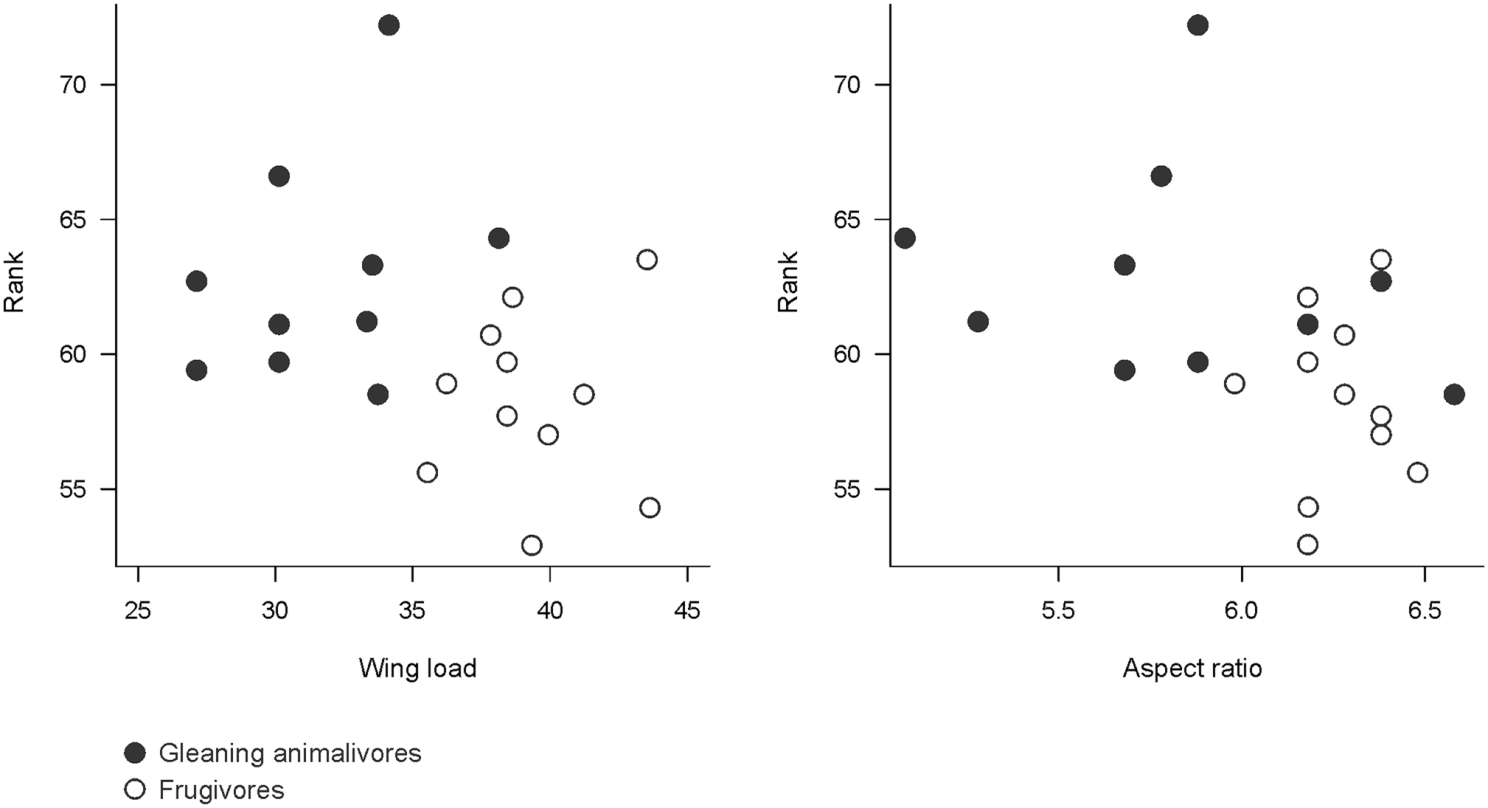
Relationship between rank values (mean number of captures weighted by vegetation clutter of each module) and wing morphology (wing load and aspect ratio) of 21 bat species captured along the BR-319 highway, Central Amazonia.

Bat assemblages of the BR-319 highway (Fig 2) showed a nested structure (NODFc, Fill = 51%, nestedness degree = 67.25, *Z* = 2.63, *P* = 0.008). The species composition in modules with more cluttered understory represented a subset of the species in the modules with more open vegetation. The module with most open vegetation (53% clutter) had twice as many species as the modules with densest vegetation (> 70% clutter). Only three species (*Trinycteris nicefori*, *Lophostoma brasiliense*, and *Vampyriscus bidens*) in modules with high clutter were not captured in the modules with more open vegetation. As vegetation clutter decreased, more species were added to the bat assemblages, especially canopy frugivorous bats, such as *Mesophylla macconelli*, *V*. *brocki*, *A*. *concolor*, *A*. *gnomus*, *A*. *obscures*, and *A*. *planirostris* (Fig 2). The rank values of abundance weighted by the vegetation clutter (Fig 2) of the animalivores was greater than for frugivorous bats (*t* test, *t* = 3.08, *P* = 0.007). Animalivorous bats also had a rank value greater than canopy frugivores (ANOVA, *F* = 4.70, *P* = 0.022, Tukey test, *P* = 0.024; Fig 2), but not significantly different from understory frugivores (Tukey test, *P* = 0.16). Rank values did not differ between canopy and understory frugivorous bats (Tukey test, *P* = 0.99). This indicates that animalivorous and understory frugivorous bats were more abundant and have higher occurrence along the vegetation clutter gradient, while canopy frugivorous bats were largely restricted to modules with more open vegetation (Fig 2). Nevertheless, the rank values (Fig 4) were not correlated with mean aspect ratio (GLM, *r* ^*2*^ = 0.17, *t* = -0.61, *P* = 0.55) or wing load (GLM, *r* ^*2*^ = 0.11, *t* = 0.92, *P* = 0.37). The aspect ratio (*t* test, *t* = 2.79, *P* = 0.018) and wing load (*t* test, *t* = 5.68, *P* < 0.0001) of the species captured were higher for frugivores than for gleaning animalivores. Wing load of the canopy and understory frugivores was higher than for gleaning animalivores (ANOVA, *F* = 15.68, *P* < 0.0001), while aspect ratio differed only between understory frugivores and animalivores (ANOVA, *F* = 4.17, *P* = 0.033). Wing characteristics did not differ significantly between canopy and understory frugivores (Tukey test, *P* > 0.88).

## Discussion

Ground-vegetation clutter influenced the structure of bat assemblages at a regional scale in our study area in Central Amazonia. The decrease in the total number of species and guilds in dense vegetation was responsible for most of the difference in bat-species composition between modules. Overall, the number of species in the modules with more cluttered vegetation (> 72%) was half that of the modules with more open vegetation (<55.6%). Reduction in the number of bat species in sites with cluttered vegetation has also been documented in other studies with phyllostomid bats [2,29,31], and for aerial insectivorous bats in temperate zones. Activity of aerial insectivorous bats is generally greater along trails, in riparian forest and along small streams where vegetation clutter is lower [4,59–63].

Some authors have suggested that species richness is mainly influenced by factors that operate on local scales [26,28]. In our study, the total number of species and guilds of phyllostomid bats was strongly associated with vegetation structure on the scale of the modules, which cover many km^2^. The vegetation structure determines the abundance of obstacles that limit bat flight, but could reflect other variables, such as food and roost availability, that also affect bat distributions. In most places, plant assemblages determine the physical structure of the vegetation [64]. Structural complexity of the vegetation and plant assemblages in the Madeira-Purus interfluve are strongly affected by groundwater and soil characteristics [65,66]. Variation in plant species composition along the soil and water gradient in the BR-319 modules may alter the fruit and insect species available for bats. Frugivorous bats are directly dependent on the distribution of food-plant species. Different genera of bats prefer fruits of specific genera. *Artibeus* bats eat mainly *Ficus* (Moraceae) and *Cecropia* (Cecropiaceae) species, and *Carollia* species eat more *Piper* fruits [67]. Sympatric *Carollia* species also tend to eat different *Piper* species [67]. *Piper* species distributions in the BR-319 were strongly associated with the soil texture gradient and two abundant species of *Piper* eaten by bats had clear changes in abundance along the soil gradient of the BR-319 [68]. For gleaning animalivores, the change in plant composition might generate an indirect effect by attracting different species of phytophagous insects. Plants support a wide array of herbivore species. In turn, herbivores feed on groups of closely related genera or plant species [69–71]. Thus, changes in plant assemblages may cause changes in composition of insect assemblages. However, if clutter is only a surrogate for some other variable, its relationship with that variable must be very strong and direct. While that is possible, we think it more likely that landscape-wide clutter is likely to be the major direct determinate of the bat assemblages in this system.

Nestedness patterns provide clues about the processes that affect species distributions among sites. Species of the modules with denser vegetation represented a subset of species in modules with more open vegetation, characteristic of a nested distribution of assemblages [61,72]. This nested pattern probably results from differences in guild distributions along the vegetation-clutter gradient. Captures of canopy frugivores were higher in modules with more open understory, while animalivores and understory frugivores were more tolerant of vegetation clutter and occurred both in open and cluttered sites.

Differences in habitat use by the guilds of phyllostomid bats have been related to the morphological characteristics of their wings and foraging behavior [8,54]. Animalivorous bats had values of aspect ratio and wing load smaller than frugivorous bats, as reported in other studies [8,54]. Lower values in animalivorous bats indicate short wings, with a large surface area and rounded tips that allow a slower and more maneuverable flight in highly cluttered environments [8,54]. These bats locate their prey passively listening for noises produced by moving arthropods on vegetation or on the ground [13,14]. When prey is located, they need agile but slow flight to capture them. In contrast, frugivorous bats tend to have long narrow wings and can fly long distances, but are less agile in narrow space. Frugivorous bats have evolved two distinct foraging strategies [16,17,73]. Canopy frugivores forage on fruit trees that are present at low densities, exhibit asynchronous fruiting within the population, and produce a great quantity of fruits for a short period. In contrast, understory frugivores are specialized on shrubs and small trees that occur at moderate densities and have more localized distributions. Shrubs produce few ripe fruits per night, but fruiting extends over weeks or months. Therefore, understory frugivores tend to forage in a relatively small area for long periods, while canopy frugivores fly long distance among several feeding patches within a night. Canopy frugivores are highly mobile and open spaces allow long-distance travel in search of trees with lower energy cost during flight [74–76]. Our results indicate that canopy frugivores were more abundant in modules with more open vegetation, while understory frugivores occurred throughout the vegetation-clutter gradient. Thus, ground-vegetation clutter predicted species and guild occurrence within assemblages.

We did not find a correlation between species distribution along the vegetation-clutter gradient, summarized by the rank values of the abundance weighted by the vegetation clutter, and wing characteristics. The values of aspect ratio and wing load of the animalivores was more variable than in frugivores (canopy and understory species), indicating higher variability in space use among animalivorous species. This may have contributed to greater variability in the rank values of animalivores species and their overlap with frugivores. Wing characteristics reflect the dynamics of bat flight in different situations. However, habitat selection in Phyllostomid bats is not uniquely determined by the wing shape, and is apparently more influenced by combinations of factors, such as diet, foraging behavior and ability to detect food in the vegetation.

Some authors have reported that frugivorous bats are more frequently captured in dense forest vegetation [1,29,77], which is in contrast to our results. However, those studies evaluated the effect of vegetation structure on bat assemblages in disturbed habitats. Secondary regrowth forests have denser vegetation than undisturbed forest [53]. However, some frugivorous bats, mainly understory species, are tolerant of disturbed habitats and increase in the abundance in secondary forests [77,78]. Frugivorous bats have good dispersal abilities and explore disturbed areas in search of fruit from pioneer plants [77,79]. Disturbed areas often have roads or edges of pasture that the bats can use for locomotion. Many species of frugivorous bats cross open areas [80], but may not traverse large deforested areas. Gleaning animalivorous bats are sensitive to habitat disturbance and disappear from small fragments and sites with constant disturbance [1,30,77]. Therefore, in anthropogenic disturbed areas, habitat selection by frugivorous and animalivorous bats appears to depend more on the habitat quality than the level of vegetation clutter.

Our results support the hypothesis that variation in vegetation structure at a local scale also structures assemblages of bats at regional scales. Ground-vegetation clutter decreases species and guild richness, with less pronounced effects on the gleaning animalivores and understory frugivores than on canopy frugivores. Moreover, foraging strategy and diet are better able to predict the spatial distribution of bats than the wing characteristics of the species alone.

Several studies have measured vegetation structure as a qualitative variable, but this is a course way to represent the vegetation-clutter gradient. Classifications of vegetation into categories may not distinguish sites with low contrasts in vegetation structure. We found strong effects of vegetation clutter, even though clutter only varied between about 40% and 70%. However, we may have been able to identify subtle landscape effects because of the standardized RAPELD sampling design [35,36], in which each sample unit is relatively homogeneous in terms of environmental gradients considered most relevant for the species distribution at the local scale. This enabled spatial and temporal comparisons with similar effort, sampling method, and design.

## Supporting Information

S1 Fig Schematic illustration of digital photographs used to quantify the understory vegetation clutter in each sample plot. A) A white cloth was tied to a 3 x 3 m aluminum frame to create a panel that would contrast with the vegetation. The panel was positioned parallel to and 8m from the mist-net. A digital camera placed beside the mist net photographed the cloth. B) Steps to measure the density of understory vegetation in the Sidelook 1.1.01 program [46]. 1) digital photo of the white panel; 2) delimitation of the white panel area; 3) transformation of the photos into black-and-white images with black areas representing the vegetation.
(TIF)

S2 Fig Species-accumulation curves for phyllostomid bats captured along the BR-319 highway. Dashed lines represent 95% confidence intervals. Open circle indicates the estimated number of species (± SD) based on the Jackknife 1 estimator.
(TIF)
