# Correction: Ground-Vegetation Clutter Affects Phyllostomid Bat Assemblage Structure in Lowland Amazonian Forest

**DOI:** 10.1371/journal.pone.0136226

**Published:** 2015-08-14

**Authors:** The PLOS ONE Staff

The legends for Figs 2 and 3 are incorrectly switched. The legend that appears for Fig 2 should appear with Fig 3, and the legend that appears for Fig 3 should appear with Fig 2. The figures appear in the correct order. The publisher apologizes for this error.

Additionally, Fig 2 is missing sections E and F. Fig 4 is missing the category “Understory Frugivores”. Please view the complete, correct Figs 2, 3 and 4, and associated legends here.

**Fig 2:**
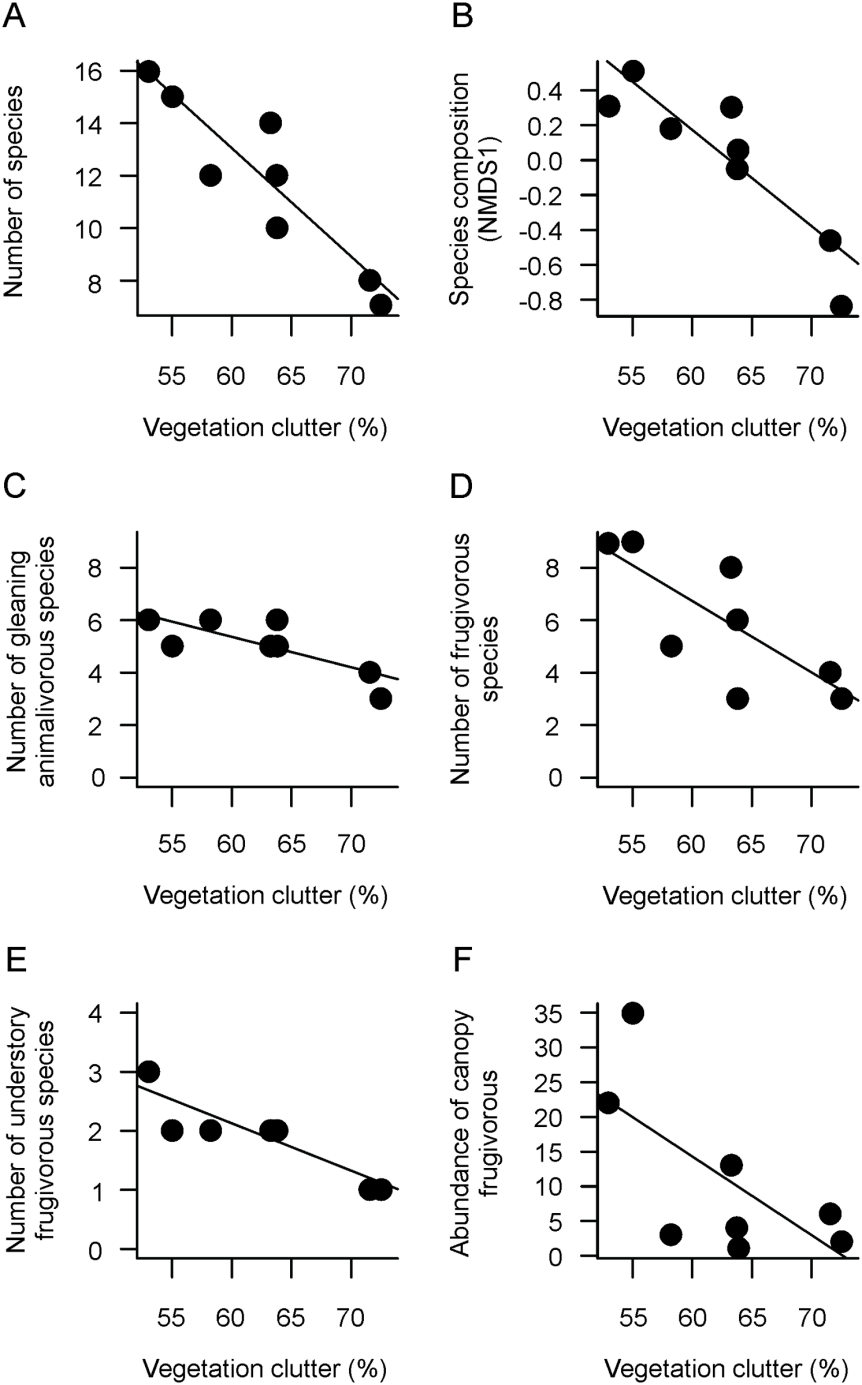
Relationships between vegetation clutter and (A) number of bat species, (B) bat-species composition summarized by the first axis of a NMDS analysis, (C) number of gleaning animalivorous species, (D) number of frugivorous species, (E) number of understory frugivorous species, and (F) abundance of canopy frugivores.

**Fig 3:**
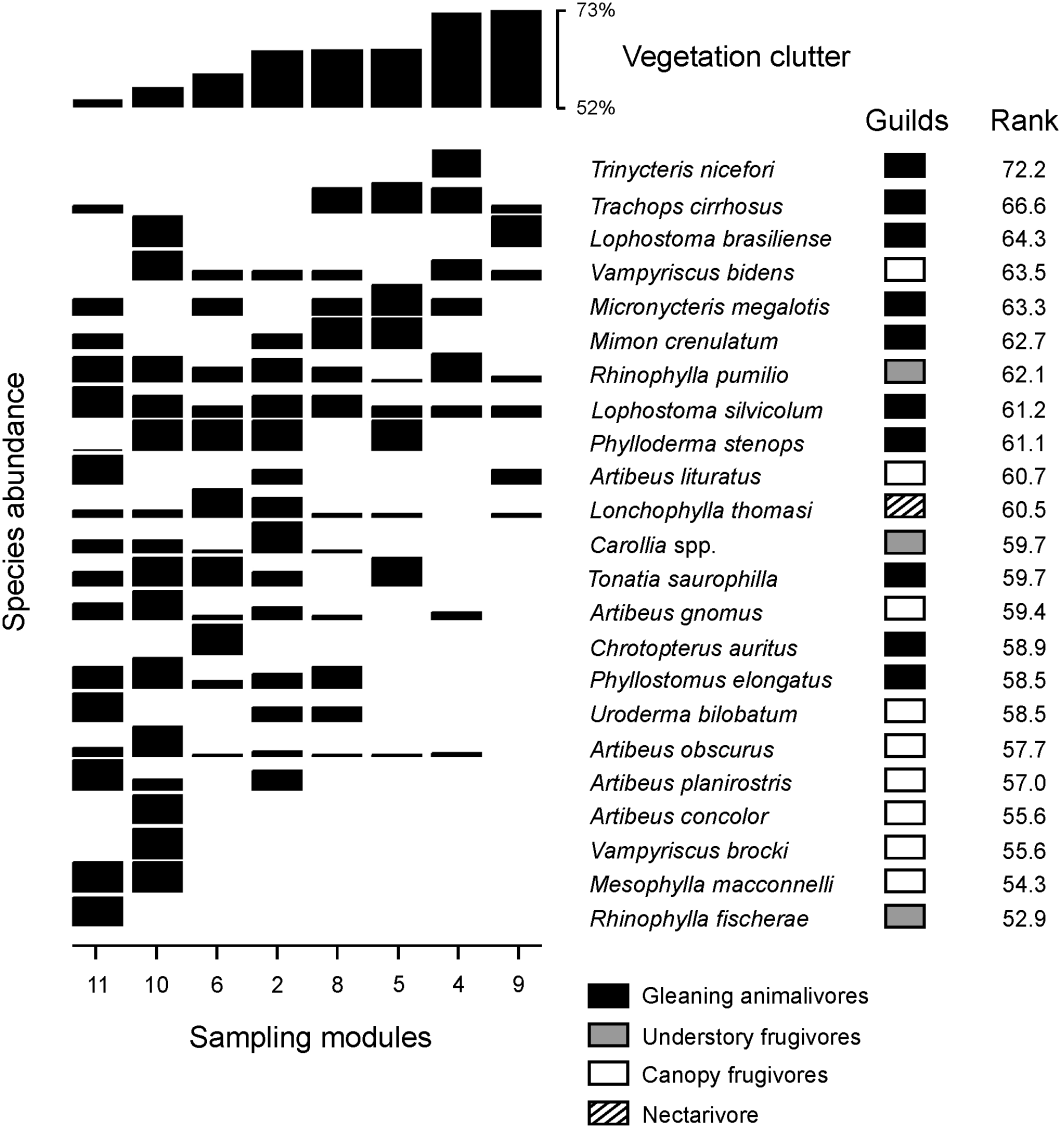
Relationship between bat abundance and the gradient of vegetation clutter. The horizontal order of the sampling modules was based on the gradient in vegetation clutter. The vertical order of species was based on the average number of captures weighted by vegetation clutter of each module, as indicated by rank values. Species with higher rank values are placed near the top of the graph. Black squares represent gleaning animalivorous bats, white squares canopy frugivores, grey squares understory frugivores, and hatched squares the nectarivore.

**Fig 4:**
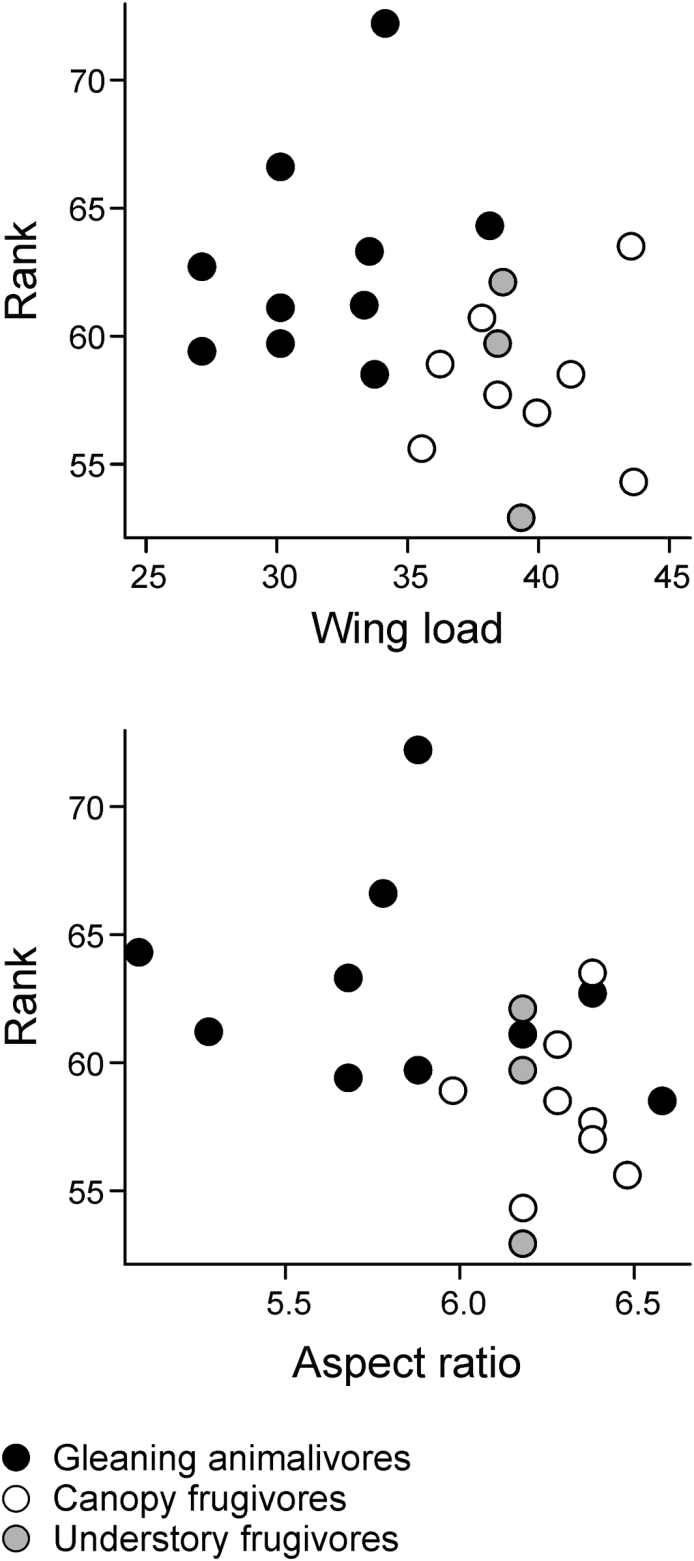
Relationship between rank values (mean number of captures weighted by vegetation clutter of each module) and wing morphology (wing load and aspect ratio) of 21 bat species captured along the BR-319 highway, Central Amazonia.
