# Supplementary figures and images for: Ground-Vegetation Clutter Affects Phyllostomid Bat Assemblage Structure in Lowland Amazonian Forest
Source: PLoS One. 2015 Jun 12;10(6):e0129560. doi: 10.1371/journal.pone.0129560 (PMC4466577; doi:10.1371/journal.pone.0129560)

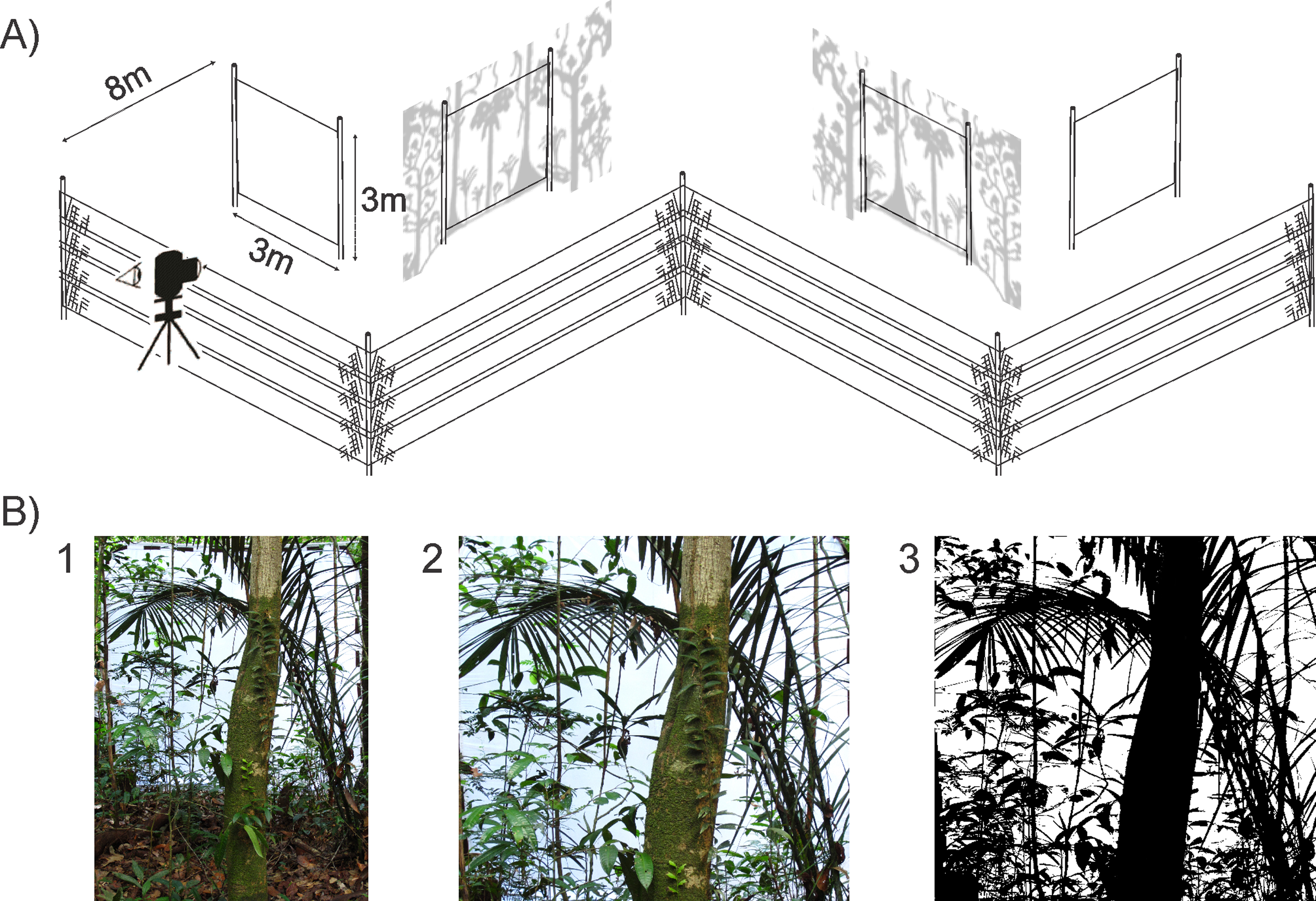

Supplement: S1 Fig — A) A white cloth was tied to a 3 x 3 m aluminum frame to create a panel that would contrast with the vegetation. The panel was positioned parallel to and 8m from the mist-net. A digital camera placed beside the mist net photographed the cloth. B) Steps to measure the density of understory vegetation in the Sidelook 1.1.01 program [46]. 1) digital photo of the white panel; 2) delimitation of the white panel area; 3) transformation of the photos into black-and-white images with black areas representing the vegetation. (TIF) [file pone.0129560.s001.tif]

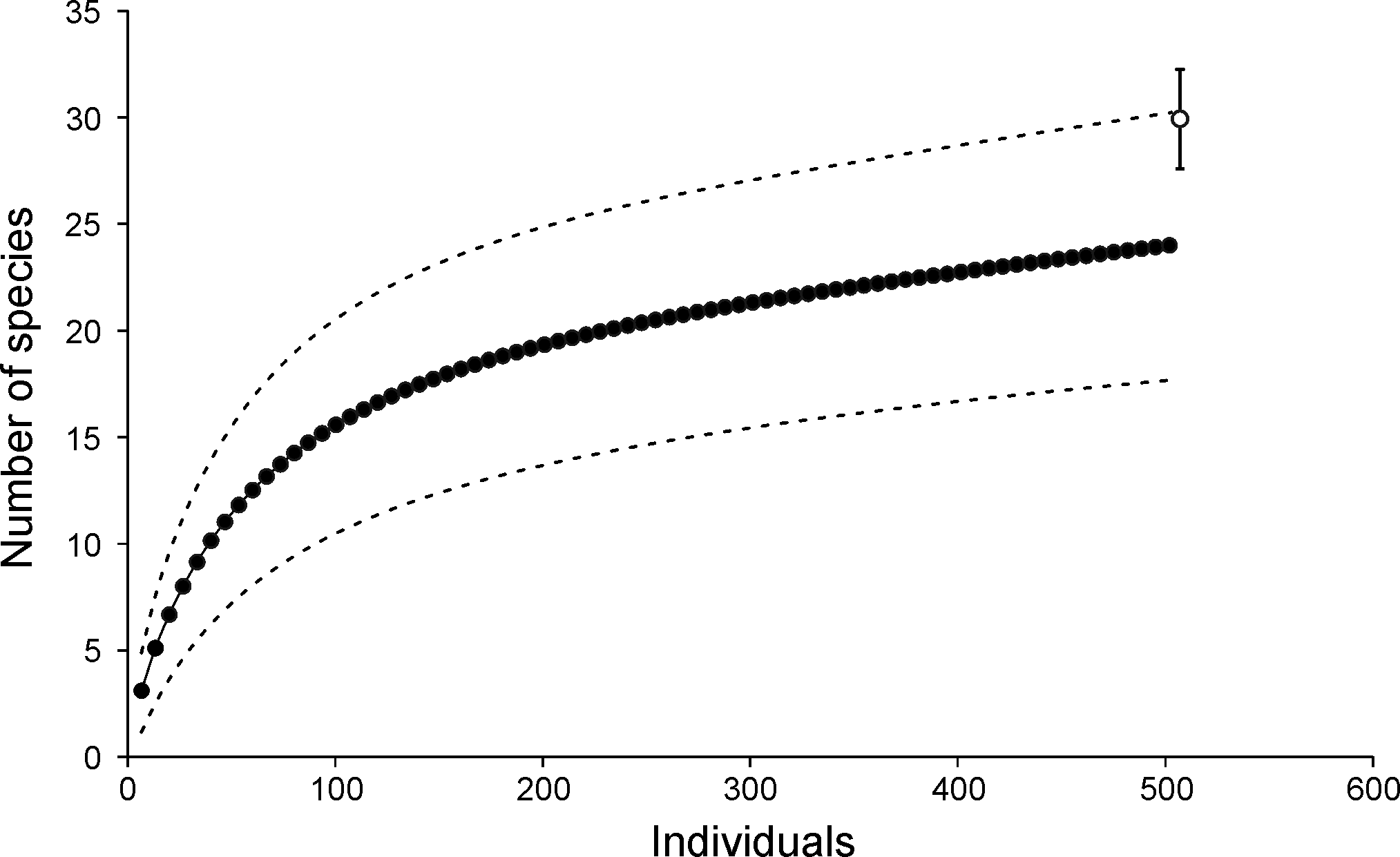

Supplement: S2 Fig — Dashed lines represent 95% confidence intervals. Open circle indicates the estimated number of species (± SD) based on the Jackknife 1 estimator. (TIF) [file pone.0129560.s002.tif]
